# Infusion of D1 Dopamine Receptor Agonist into Medial Frontal Cortex Disrupts Neural Correlates of Interval Timing

**DOI:** 10.3389/fnbeh.2015.00294

**Published:** 2015-11-10

**Authors:** Krystal L. Parker, Rafael N. Ruggiero, Nandakumar S. Narayanan

**Affiliations:** Neurology, University of IowaIowa City, IA, USA

**Keywords:** medial frontal cortex, dopamine, Parkinson's disease, interval timing, cognition

## Abstract

Medial frontal cortical (MFC) dopamine is essential for the organization of behavior in time. Our prior work indicates that blocking D1 dopamine receptors (D1DR) attenuates temporal processing and low-frequency oscillations by MFC neuronal networks. Here we investigate the effects of focal infusion of the D1DR agonist SKF82958 into MFC during interval timing. MFC D1DR agonist infusion impaired interval timing performance without changing overall firing rates of MFC neurons. MFC ramping patterns of neuronal activity that reflect temporal processing were attenuated following infusion of MFC D1DR agonist. MFC D1DR agonist infusion also altered MFC field potentials by enhancing delta activity between 1 and 4 Hz and attenuating alpha activity between 8 and 15 Hz. These data support the idea that the influence of D1-dopamine signals on frontal neuronal activity adheres to a U-shaped curve, and that cognition requires optimal levels of dopamine in frontal cortex.

## Introduction

Frontal dopamine signaling is crucial for memory and cognition (Goldman-Rakic, 1998). Dysfunction of medial frontal dopamine is involved in diseases such as ADHD, schizophrenia, and Parkinson's disease (Cools et al., 2001; Abi-Dargham et al., 2002; Bellgrove et al., 2005; Narayanan et al., 2013a). Cognition requires optimal levels of frontal dopamine signaling (Cools and D'Esposito, 2011). There are two major classes of dopamine receptors: D1 and D2. Of these, D1-type dopamine receptors (D1DR) have been specifically implicated in cognition (Goldman-Rakic et al., 2004; Kim et al., 2015). Both agonists and antagonists of D1DRs impair neural correlates of cognitive processes such as working memory and attention (Williams and Goldman-Rakic, 1995; Granon et al., 2000; Vijayraghavan et al., 2007).

Here, we study how manipulating D1DRs influences neural activity during timing tasks. Timing is an executive function that requires working memory for temporal rules as well as attention to the passage of time (Parker et al., 2013a; Merchant and de Lafuente, 2014). Timing is well-suited to study human diseases of cognition because it involves common dopamine-dependent frontal cortical mechanisms in humans and rodents (Merchant et al., 2008; Narayanan et al., 2013b; Parker et al., 2013a, 2015; Merchant and de Lafuente, 2014). We focus on the MFC because rodents lack lateral frontal regions and because of MFC's homologies between rodents and humans (Laubach, 2011). Blocking D1DRs in the MFC impairs performance on interval timing tasks, in which subjects initiate a motor response several seconds after an instructional cue (Narayanan et al., 2012). In MFC, there are two prominent neuronal correlates of temporal processing: (1) ramping activity, or neuronal firing that consistently changes over the temporal interval (Durstewitz, 2003; Kim et al., 2013; Parker et al., 2014; Xu et al., 2014) and (2) low-frequency oscillations around ~4 Hz triggered by the instructional stimuli (Parker et al., 2014, 2015). Blocking D1DRs attenuates both ramping activity and attenuates ~4 Hz oscillationsHz (Parker et al., 2014). It is unclear how these two correlates of medial frontal cortex activity are influenced by MFC D1DR agonists. Previous data demonstrating that D1DR agonists impair cognitive performance and neuronal correlates of cognition in frontal cortex predict that D1DR agonists should attenuate both ramping activity and ~4 Hz oscillations.

To test this idea, we recorded neural activity and field potentials from MFC following infusion of SKF82958 (Gilmore et al., 1995), a D1DR agonist, into MFC of animals performing an interval timing task. We report that focal infusion of D1DR agonist attenuates MFC ramping neuronal activity and increases cue-dependent delta activity. These data support the idea that temporal processing by single neurons, like mnemonic processing, requires optimal levels of frontal D1DR signaling.

## Materials and methods

Seven male Long-Evans rats (aged 2 months; 200–225 g) were trained to perform an interval timing task. Animals were motivated by water restriction, while food was available *ad libitum*. Rats consumed 10–15 mL of water during each behavioral session and additional water (5–10 mL) was provided 1–3 h after each behavioral session in the home cage. Single housing and a 12 h light/dark cycle were used; all experiments took place during the light cycle. Rats were maintained at ~90% of their free-access body weight during the course of these experiments and received one day of free access to water per week. All procedures were approved by the Animal Care and Use Committee at the University of Iowa.

**Figure 1:**
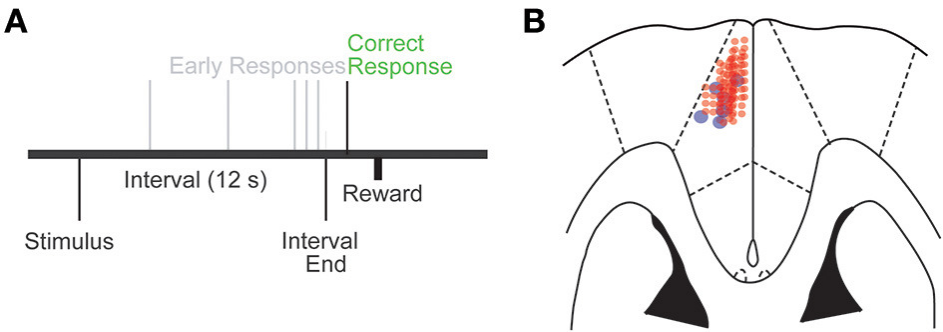
**(A)** Interval timing task. Subjects estimate a 12 s interval starting with the onset of a discriminative stimulus by making a motor response; multiple responses per trial are permitted. **(B)** Cannula locations in the MFC were reconstructed from histological sections; cannulae are marked by blue circles, and electrode locations by red circles.

Rats were trained in interval timing tasks using standard operant procedures described in detail previously (Parker et al., 2014, 2015). First, animals learned to make operant lever presses to receive liquid rewards. After fixed-ratio training, animals were trained in a 12 s fixed-interval timing task in which rewards were delivered for responses after a 12 s interval (Figure 1A). Rewarded presses were signaled by a click and an “off” houselight. Each rewarded trial was followed by an intertrial interval of 6–12 s, randomly chosen. Intertrial intervals concluded with an “on” houselight signaling the beginning of the next trial. Early responses occurring before 12 s were not reinforced. The houselight was turned on at trial onset and lasted until the onset of the intertrial interval.

Interval timing performance was evaluated by the timing efficiency or the number of lever presses occurring in the 11–12 s, timed interval in comparison to all lever presses occurring from 1 to 12 s of the trial. Curvature was also used to evaluate performance during the interval timing task. Curvature indexes increase as animals' responses are guided by time and measure the deviation from the cumulative response record of a straight line (Fry et al., 1960; Parker et al., 2015). Curvature of time-response histograms is a robust measure of animals' timing as it is independent of response rate as responses are controlled by time (Caetano and Church, 2009; Parker et al., 2014).

Rats trained in the 12 s interval timing task were implanted with a microwire electrode array and a 33-gauge infusion cannula (Plastics One) in the MFC according to procedures described previously (Parker et al., 2014). Briefly, animals were anesthetized using Ketamine (100 mg/kg) and Xylazine (10 mg/kg). A surgical level of anesthesia was maintained with ketamine supplements (10 mg/kg). Under aseptic surgical conditions, the scalp was retracted, and the skull was leveled between bregma and lambda. A single craniotomy was drilled over the area above the MFC and four holes were drilled for skull screws. A microelectrode array was implanted in MFC (coordinates from bregma: AP: +3.2, ML ± 1.2, DV −3.5 @ 12° in the lateral plane). Electrode ground wires were wrapped around the skull screws. The infusion cannula was then lowered to target the neurons being recorded (coordinates from bregma: AP: +0.3, ML ± 1.0, DV −4.6 @ 40° in the lateral plane; targeting bregma coordinates AP +3.2, ML ± 1.0, DV −3.4 in the center of the recording array). The craniotomy was sealed with cyanoacrylate (“SloZap,” Pacer Technologies, Rancho Cucamonga, CA) accelerated by “ZipKicker” (Pacer Technologies), and methyl methacrylate (i.e., dental cement; AM Systems, Port Angeles, WA). Following implantation, animals recovered for one week before being reacclimatized to behavioral and recording procedures.

At the beginning of recording experiments, rats received a saline infusion into the MFC ~45 min prior to neurophysiological recording according to procedures described previously (Parker et al., 2013b, 2014). On the subsequent day rats received an infusion of the D1DR agonist SKF82958 into the MFC. Infusion was conducted by inserting an injector into the guide cannula and 0.5 μL of infusion fluid was delivered at a rate of 30 μL/h (0.5 μL/min) via a syringe infusion pump (KDS Scientific, Holliston, MA). After injections were complete, the injector was left in place for 2 min to allow for diffusion. Statistical comparisons between saline and MFC SKF82958 infusion sessions made no assumption that identical neurons were recorded on subsequent days.

Neuronal ensemble recordings in the MFC were made using a multi-electrode recording system (Plexon, Dallas, TX). Putative single neuronal units were identified on-line using an oscilloscope and audio monitor. The Plexon off-line sorter was used to analyze the signals after the experiments and to remove artifacts. Spike activity was analyzed for all cells that fired at rates above 0.1 Hz. Statistical summaries were based on all recorded neurons. No subpopulations were selected or filtered out of the neuron database. Wide-band signals were recorded using wide-band boards with bandpass filters between 0.07 and 8000 Hz and sampled at 40,000 Hz. Principal Component Analyses (PCA) and waveform shapes were used for spike sorting. Single units were identified as having (1) consistent waveform shape, (2) separable clusters in PCA space, (3) a consistent refractory period of at least 2 ms in interspike interval histograms, and (4) consistent firing rates around behavioral events (as measured by a runs test of firing rates across trials around behavioral events; neurons with |z| scores > 4 were considered “nonstationary” and were excluded). Analysis of neuronal activity and quantitative analysis of basic firing properties were carried out using NeuroExplorer (Nex Technologies, Littleton, MA), and with custom routines for MATLAB. Peri-event rasters and average histograms were constructed around light on, lever release, lever press, and lick. Microwire electrode arrays were comprised of 16 electrodes. In each animal, one electrode without single units was reserved for local referencing, yielding 15 electrodes per rat. Local field potentials (LFPs) were recorded from 4 of these electrodes per rodent. LFP channels were analog filtered between 0.7 and 100 Hz online, sampled at 1000 Hz and recorded in parallel with single unit channels using a wide-band board. Consistent with our prior work, although examples of individual neurons are shown under different drug conditions (control and MFC D1DR agonist), our statistical analyses assume that these populations of neurons are independent (Narayanan and Laubach, 2006, 2008; Narayanan et al., 2013b).

We defined ramping activity as firing rate that progressed uniformly over the interval. We quantified this in two ways: linear regression and PCA. Ramping neurons are described as those with a significant relationship of firing rates over trials vs. time in a linear regression model (Kim et al., 2013). For regression, firing rates were binned into 2 s bins. Secondly, PCA was used to identify dominant patterns of neuronal activity using orthogonal basis functions from peri-event histograms during the 12 s interval (Paz et al., 2005; Narayanan and Laubach, 2009; Bekolay et al., 2014; Narayanan and Laubach, 2014). All neurons from 6 animals per session (control and MFC D1DR agonist sessions) were included in PCA, and the first 500 ms of the interval was excluded due to stimulus-related activity. The same principal components were projected onto control and MFC SKF sessions, and the weights were compared via a *t*-test (Chapin and Nicolelis, 1999; Narayanan and Laubach, 2014).

Time-frequency calculations were computed using custom-written Matlab routines (Cavanagh et al., 2009). Time-frequency measures were computed by multiplying the fast Fourier transformed (FFT) power spectrum of LFP data with the FFT power spectrum of a set of complex Morlet wavelets (defined as a Gaussian-windowed complex sine wave: $e^{i2\pi tf}e^{-\frac{t^{2}}{2x\sigma^{2}}},$ where *t* is time, f is frequency (which increased from 1 to 50 Hz in 50 logarithmically spaced steps), and defines the width (or “cycles”) of each frequency band, set according to 4/(2πf)), and taking the inverse FFT. The end result of this process is identical to time-domain signal convolution, and it resulted in: (1) estimates of instantaneous power (the magnitude of the analytic signal), defined as Z[t] (power time series: p(t) = real[z(t)]^2^ + imag[z(t)]^2^); and, (2) phase (the phase angle) defined as = arctan(imag[z(t)]/real[z(t)]). Each epoch was then cut in length surrounding the event of interest (−500 to +2000 ms). Power was normalized by conversion to a decibel (dB) scale (10^*^log10[power(t)/power(baseline)]) from a pre-stimulus baseline of −500 to −300 ms, allowing a direct comparison of effects across frequency bands. Statistical significance was computed via a paired *t*-test comparing saline and D1DR agonist sessions in 3 three frequency bands, delta (1–4 Hz), theta (4–8 Hz), and alpha (8–15 Hz).

When experiments were complete, rats were sacrificed by injections of 100 mg/kg sodium pentobarbital, and transcardially perfused with 10% formalin. Brains were post fixed in a solution of 10% formalin and 20% sucrose before being sectioned on a freezing microtome. Brain slices were mounted on gelatin-subbed slides and stained for cell bodies using DAPi. Histological reconstruction was completed using post mortem analysis of electrode and cannula placements and confocal microscopy in each animal. These data were used to determine electrode and cannula placement within the MFC.

## Results

**Figure 2:**
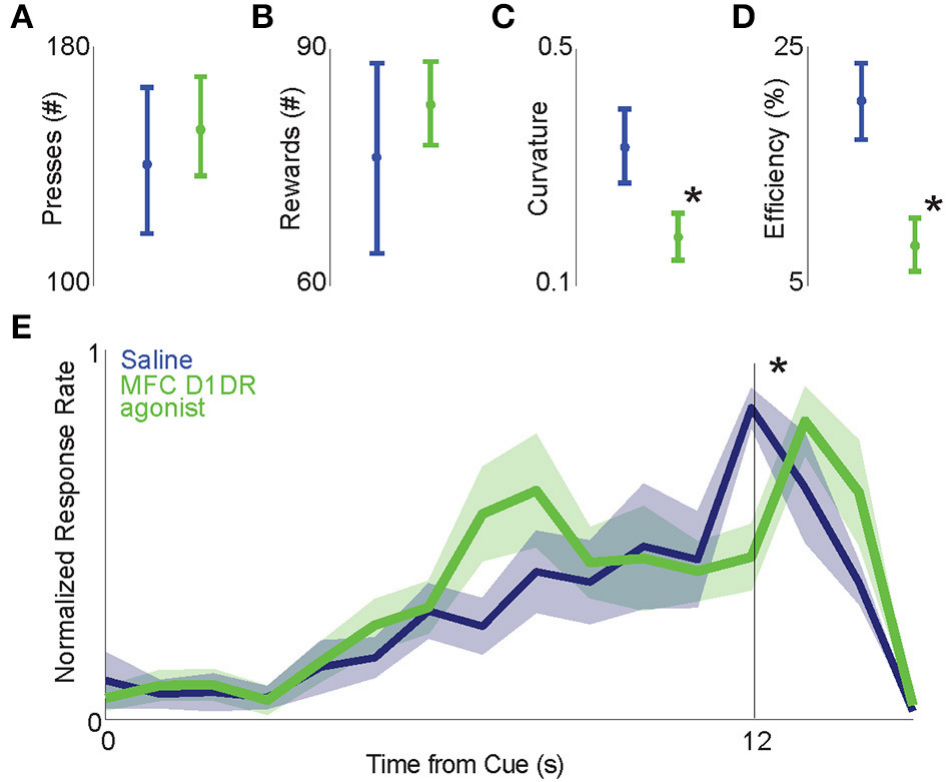
**Focal infusion of MFC D1 agonist into the MFC decreases the efficiency of interval timing without changing other aspects of behavior**. MFC D1DR agonists (green) did not change the number of overall lever presses **(A)** or the number of rewards **(B)** when compared to saline sessions (blue). **(C)** Curvature indices of time-response histograms we significantly reduced in MFC D1DR agonist sessions compared to control sessions, and **(D)** MFC D1DR agonist decreased how efficiently animals timed. **(E)** Time-response histograms during interval timing sessions show less accurately timed lever pressing in animals following infusion of MFC D1DR agonist (in 7 animals). Asterisks represent significance at *p* < 0.05 via a *t*-test.

We trained seven rats to perform an interval timing task and implanted recording electrodes and an infusion cannula into the MFC (Figures 1A,B). After recovery from surgery, we focally infused the D1DR agonist SKF82958 into the MFC (MFC D1DR agonist sessions) prior to neuronal recordings during interval timing tasks. Compared to saline sessions, rodents had similar numbers of lever presses in MFC D1DR agonist infusion sessions (153.2 ± 16.8 vs. 141.5 ± 24.7 in saline sessions; Figure 2A), and acquired similar numbers of rewards (82.8 ± 5.3 vs. 75.8 ± 12.1 in saline sessions Figure 2B). MFC D1DR agonist infusion significantly decreased how efficiently animals responded at interval end [% of responses between 11 and 12 s; 0.08 ± 0.2 vs. 0.20 ± 0.03 in saline sessions; *t*_(5)_ = 3.4, *p* < 0.02; Figure 2D]. Interval timing performance was measured using a curvature index where a higher curvature corresponds to higher deviations from a straight line (24). Here, we found a flatter curvature of time-response histogram in MFC D1DR agonist sessions in comparison to saline sessions [0.23 ± 0.04 vs. 0.32 ± 0.03; *t*_(5)_ = 2.63, *p* < 0.05; Figure 2C]. Taken together, these data indicate that focal MFC D1DR agonist decreases how efficiently animals guide their responses in time without changing lever pressing or motivation (Figure 2E).

In seven animals, we recorded 41 neurons in sessions with MFC D1DR agonist, and 47 neurons in saline sessions. Focal MFC SKF82958 infusion did not change overall firing rate (2.9 ± 0.5 vs. 3.6 ± 0.9 Hz in saline sessions). We identified stimulus-related neurons and neurons related to lever pressing by paired t-tests of firing rate before and after stimulus and lever press (Parker et al., 2014). By these criteria, similar fractions of MFC neurons were stimulus-related in MFC D1DR agonist sessions compared to saline sessions (stimulus: 3 vs. 4 in saline sessions; press: 5 vs. 4 in saline sessions). These results provide evidence that SKF82958 did not change the basic neuronal properties of MFC.

**Figure 3:**
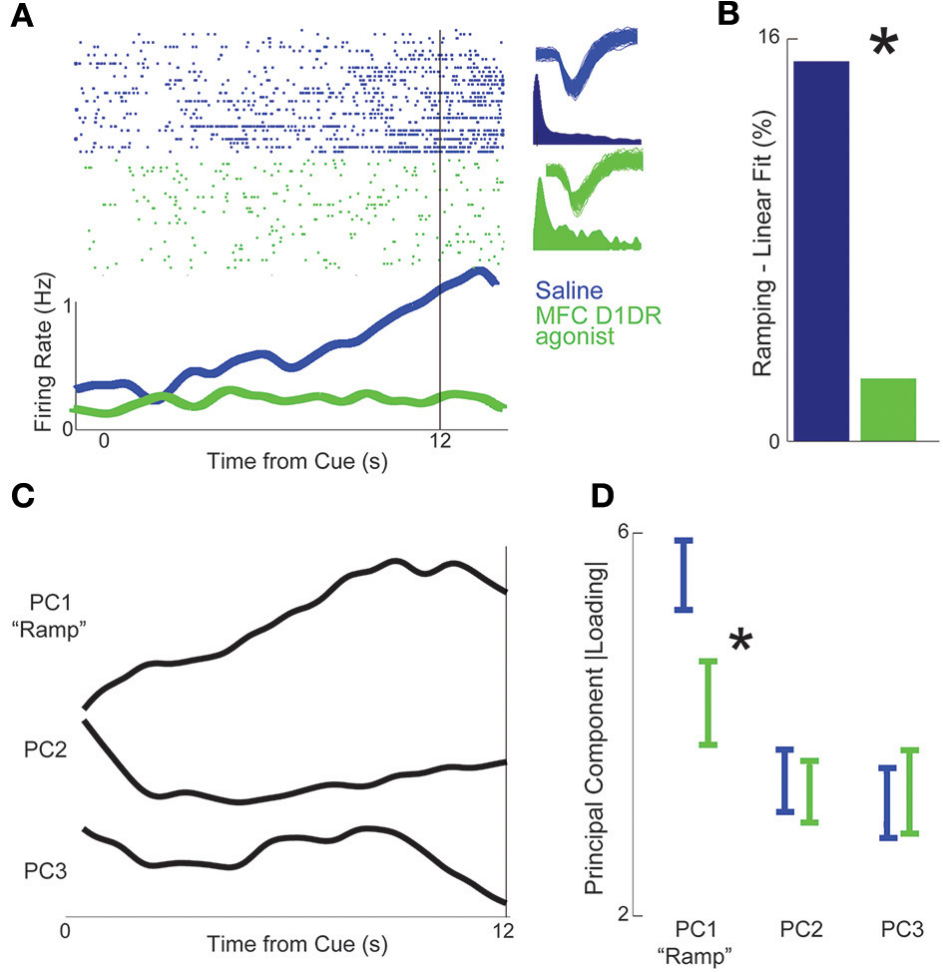
**MFC D1DR agonist attenuates ramping activity. (A)** Example of an MFC ramping neuron recorded in control (blue) sessions. In green, the same putative neuron from the control session is shown in MFC D1DR agonist sessions. Top plot is a raster plot depicting the activity of a single neuron selected from each condition. Each row is a trial from the experiment and each dot is an action potential from a single neuron. **(B)** There was significantly less ramping activity in MFC D1DR agonist sessions, as identified by the number of neurons with a significant linear fit via regression. Statistical comparisons assumed that independent populations were recorded in control and D1DR blockade sessions. **(C)** Principal component analysis in control sessions revealed that ramping activity was the most prominent pattern of neural activity among MFC neurons (PC1). **(D)** To directly compare ramping activity, we projected PCs from control sessions onto MFC D1 agonist sessions. PC1 explained significantly less variance in MFC D1DR agonist sessions, while PC2 and 3 were unchanged. Asterisk represents significance at *p* < 0.05 via a *t*-test. All statistics treated each session independently. Taken together, these data suggest that MFC D1DR agonist attenuates ramping activity of neurons.

Ramping activity, or neuronal activity that consistently increases or decreases over a temporal interval, is a key correlate of temporal processing in MFC (Figure 3A) (Durstewitz, 2003; Kim et al., 2013). Our recent work has shown that MFC D1DR blockade attenuates ramping activity (Parker et al., 2014). Here we investigated how MFC D1DR agonists influence ramping activity. We identified ramping activity using linear regression to identify neurons with a significant linear fit. Only 1 neuron had a significant linear fit in MFC D1DR agonist sessions, compared with 7 neurons in saline sessions (2 vs. 15% in saline sessions; χ^2^ = 4.3, *p* < 0.04; Figure 3B). When neural data was shuffled in time, no significant differences in ramping neurons were observed (3 neurons/6% in saline sessions vs. 2 neurons/5% in SKF; χ^2^ = 0.09, *p* < 0.76). Ramping activity is also readily identified by principal component analysis, in which ramping components are PC1 (Narayanan and Laubach, 2009; Bekolay et al., 2014; Parker et al., 2014). Consistent with prior work, ramping activity was PC1 and explained 28% of variance among neuronal ensembles in saline sessions (Figure 3C). PC1 loaded more significantly onto saline sessions compared to MFC D1DR agonist sessions [Figure 3D; *t*_(86)_ = 2.3, *p* < 0.03]. Taken together, these data indicate that MFC D1DR agonists attenuated ramping activity in MFC.

**Figure 4:**
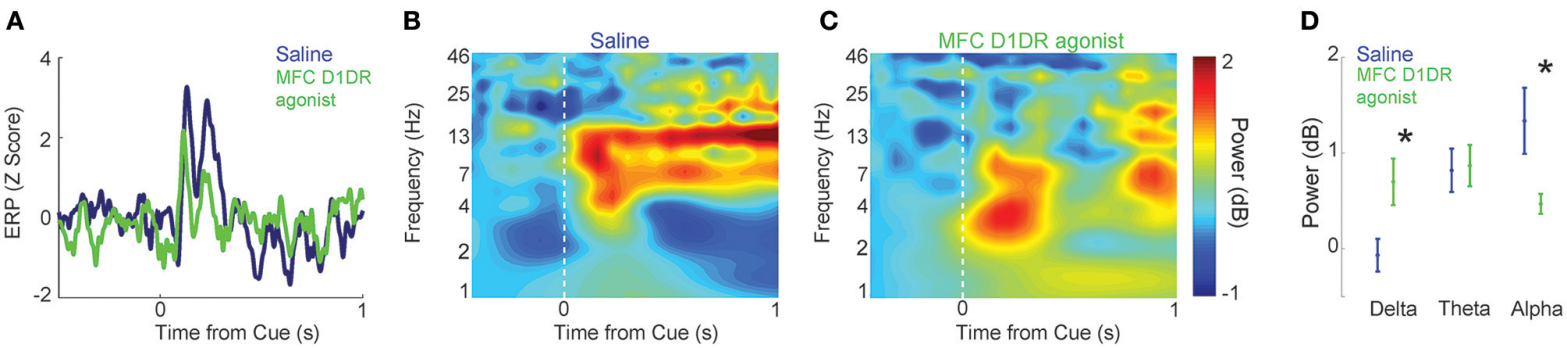
**MFC D1DR agonists influence oscillatory patterns of MFC LFP during interval timing tasks. (A)** Event-related potentials from all LFP channels in 7 rodents (17 channels) revealed a stimulus-triggered peak the MFC after stimulus onset. MFC ERP is unaffected by MFC D1DR agonists. **(B)** Time-frequency analysis revealed a burst of activity from 4 to 15 Hz following the onset of the cue. **(C,D)** Infusions of MFC D1DR agonist significantly increased delta activity between 1 and 4 Hz and attenuated alpha activity between 8 and 15 Hz while theta activity was unaffected. Asterisk represents significance at *p* < 0.05 via a *t*-test.

During timing tasks, there is a burst of ~4 Hz field potential activity after the instructional stimulus during timing tasks (Parker et al., 2014, 2015). This activity is dependent on MFC dopamine and attenuated with D1DR blockade (Parker et al., 2014, 2015). We examined how this activity changed in MFC D1DR agonist sessions among 17 LFP channels across 7 animals. Consistent with prior work in saline sessions, the instructional stimulus was followed by a burst of delta (1–4 Hz), theta (4–8 Hz) and alpha (8–15 Hz) activity (Figures 4A,B). In MFC D1DR agonist sessions, delta activity increased [*t*_(16)_ = 2.7, *p* < 0.02; 1–4 Hz 0–1 s after the cue], theta activity did not change (4–8 Hz 0–1 s after the cue), and alpha activity was attenuated [*t*_(16)_ = 3.1, *p* < 0.01; 8–12 Hz 0–1 s after the cue], when compared to saline sessions (Figures 4C,D). Taken together, these data indicate that D1DR agonist SKF82958 in MFC disrupts performance of interval timing tasks as well as neuronal correlates of temporal processing in MFC.

## Discussion

In the present manuscript, we recorded MFC neuronal ensembles while focally infusing the D1DR agonist SKF82958 into MFC during performance of an interval timing task. We found that MFC SKF82958 infusion decreased the efficiency of animals' responses during interval timing, attenuated MFC ramping activity, and altered MFC field potentials by enhancing delta activity between 1 and 4 Hz and attenuating alpha activity between 8 and 15 Hz. These data, in combination with our prior work, suggest that optimal frontal D1DR signaling is central to the temporal control of action (Parker et al., 2013b, 2014, 2015).

Combined with our work showing that MFC D1DR blockade disrupts MFC activity, our result is consistent with the idea that fluid cognition requires optimal dopamine in frontal cortex (Goldman-Rakic, 1998). This “U-shaped curve” has been shown for D1DR signaling for cognitive tasks in rats and primates (Zahrt et al., 1997; Granon et al., 2000; Vijayraghavan et al., 2007). In particular, both performance of working memory tasks as well as mnemonic delay activity of frontal neurons requires optimal frontal D1DR (Vijayraghavan et al., 2007). Optimal levels of dopamine are critical for efficient neural transmission in frontal cortex (Kroener et al., 2009).

We extend this line of work to two neuronal correlates of interval timing: ramping activity and low-frequency oscillations. Ramping activity encodes temporal processing in parietal, temporal, and frontal brain regions (Reutimann et al., 2004; Janssen and Shadlen, 2005; Narayanan and Laubach, 2009; Kim et al., 2013). Our results suggest that ramping activity predicts response time on a trial-by-trial basis (Parker et al., 2014) and like delay-activity during working memory tasks, depends on optimal MFC D1DR dopamine signaling. With both MFC D1DR antagonist SCH23390 and MFC D1DR agonist SKF82958, ramping activity is broadly attenuated, clearly demonstrating that ramping signals in MFC adhere to the same U-shaped curve as mnemonic activity during working memory tasks from primate lateral frontal cortex (Goldman-Rakic et al., 2004; Vijayraghavan et al., 2007). It is unclear, however, why ramping activity is attenuated. During timing tasks, dopamine neurons phasically fire to reward-predictive stimuli at the beginning of the task (Kobayashi and Schultz, 2008). However, endogenous, tonic dopamine levels can also affect frontal function (Kroener et al., 2009; Cools and D'Esposito, 2011). Future studies will manipulate dopamine neurons while recording from frontal neuronal ensembles to address this issue.

While ramping activity adheres to a U-shaped curve, dopamine's influence on spectral properties of MFC field potentials is more complex (Table 1). Delta activity was increased with MFC D1 agonists, while alpha was attenuated and theta was unchanged. MFC ramping neurons are coherent with ~4 Hz activity (Narayanan et al., 2013b; Parker et al., 2014). In this study, delta activity was increased but ramping activity did not improve, indicating that coupling between ramping neurons and ~4 Hz activity might also depend on optimal D1DR signaling. Our data suggest that only alpha activity strictly follows a U-shaped curve (Figure 3; Table 1). Alpha activity in frontal cortex has been associated with top-down processing (Sauseng et al., 2005), and the decreased alpha we see in this study may be reflective of decreased executive control in MFC D1DR agonist sessions.

**Table 1:** **Summary of MFC spectral activity in delta, theta and alpha bands 0–1 s after cue onset**.

	**Delta**	**Theta**	**Alpha**
Saline	−0.07 ± 0.17	0.81 ± 0.22	1.15 ± 0.29
SCH	0.32 ± 0.13	−0.11 ± 0.30	−0.10 ± 0.33
SKF	0.24 ± 0.70	0.82 ± 0.22	0.50 ± 0.09

*SCH data are extracted from previously published dataset in Parker et al. (2014)*.

Low-frequency oscillations are a key mechanism of cognitive control (Cavanagh and Frank, 2014). Rodents and humans have common low-frequency activity in delta, theta, and alpha bands during cognitive tasks (Narayanan et al., 2013b; Parker et al., 2015). These spectral activities require MFC dopamine (Parker et al., 2015). Because low-frequency oscillations can be readily observed with scalp-EEG, finding that they are sensitive to optimal frontal dopamine are of particular translational significance. In humans with Parkinson's disease, frontal dopamine may be facilitated early in the disease and profoundly influenced by the treatment (Cools et al., 2001, 2010). Several human diseases, such as schizophrenia, ADHD, and drug addiction (Abi-Dargham et al., 2002; Heijtz et al., 2007) involve dysfunctional frontal dopamine signaling. Our findings predict that in human diseases with disrupted frontal D1DR dopamine signaling, spectral activity in MFC will also be disrupted (Parker et al., 2015).

These data involve several limitations. First, we administered a pharmacological agonist with a complex receptor profile (Gilmore et al., 1995). Dopamine signaling is complex and depends on state, history, and network properties (Seamans and Yang, 2004). MFC D1DR agonists also likely cause rapid receptor internalization (Ryman-Rasmussen et al., 2005). D1DR are G-Protein receptors with diverse intracellular targets via cAMP (Kim et al., 2015). D1DR signaling can potentiate inputs of local cortical networks onto pyramidal neurons (Yang and Seamans, 1996). Thus, the functional outcome of MFC D1DR agonists on neuronal activity is quite complex. When specifically stimulating frontal neurons expressing D1DRs, interval timing performance is not disrupted but slightly enhanced (Narayanan et al., 2012). Future studies will explore the detailed action of D1DRs signaling aimed at new therapies for human diseases.

## Funding

This work was funded by The National Institute of Neurological Disorders and Stroke R01 NS078100/K08 NS078100, The National Institute of Mental Health, NARSAD Brain and Behavior Foundation, and grant #2014/22817-1, São Paulo Research Foundation (FAPESP).

### Conflict of interest statement

The authors declare that the research was conducted in the absence of any commercial or financial relationships that could be construed as a potential conflict of interest.

## Abbreviations

MFC: Medial frontal cortex
D1DR: D1 dopamine receptors
PD: Parkinson's disease.
